# Infection of a Ventricular Septal Defect Patch with *Acremonium* Species

**DOI:** 10.1155/2018/3057463

**Published:** 2018-06-25

**Authors:** Nosheen Nasir, Kauser Jabeen, Joveria Farooqi, Binish Arif Sultan, Afia Zafar, Aamir Hameed Khan, Muneer Amanullah, Farheen Ali

**Affiliations:** ^1^Department of Medicine, Aga Khan University, Karachi, Pakistan; ^2^Department of Pathology and Microbiology, Aga Khan University, Karachi, Pakistan; ^3^Department of Surgery, Aga Khan University, Karachi, Pakistan

## Abstract

A ventricular septal defect (VSD) patch infection with *Acremonium* species isolated from vegetation and blood culture is described. Antifungal treatment was discontinued after 3 months and patient developed relapse. Surgery with prolonged oral voriconazole was instituted with recovery. We emphasize importance of surgery and prolonged therapy to treat such infections.

## 1. Background

*Acremonium* species are present in the environment and mostly cause superficial and locally invasive infections after traumatic inoculation. Invasive infections are rare and are mainly reported in immunocompromised patients. It has been reported to cause osteomyelitis, sinusitis, arthritis, peritonitis, and less frequently central nervous system infections [1]. Nosocomial and postsurgical invasive infections have also been described, in particular *Acremonium* fungemia associated with central line infection and pacemaker infection [2, 3]. As invasive infections with *Acremonium* species are rare, reliable diagnosis and optimal management of these infections is challenging. Additionally, the species of *Acremonium* are morphologically very similar to each other, and thus their exact identification is not always possible despite use of modern DNA based methods. Hence, in most case reports, the agent is reported only as an *Acremonium* sp. [4]. Therapy too is challenging as high MICs against all systemic antifungals used to treat invasive mycoses have been reported [5]. Due to inherent resistance and variable clinical response to antifungals, poor outcome generally is reported in invasive *Acremonium* infections. Anecdotal evidence suggests a combination of surgical intervention, when possible, and amphotericin B for a favorable outcome [5]. Reports describing cardiac infections are even rarer. We are reporting a case of a 21-year-old male with an infected ventricular septal defect (VSD) patch, after Tetralogy of Fallot repair, with *Acremonium* species associated with fungemia and septic emboli to the lung, kidneys, liver, and spleen.

## 2. Case Report

**Figure 1:**
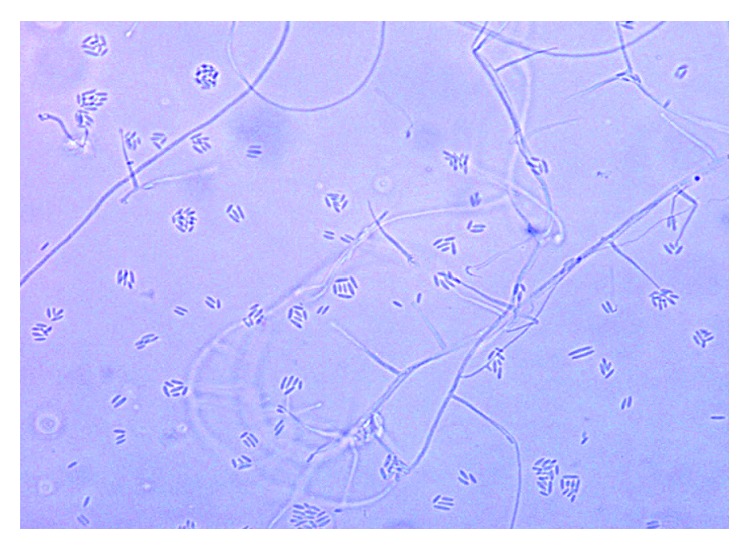
Microscopic image of the slide culture of the isolate from the prosthetic valve.

**Figure 2:**
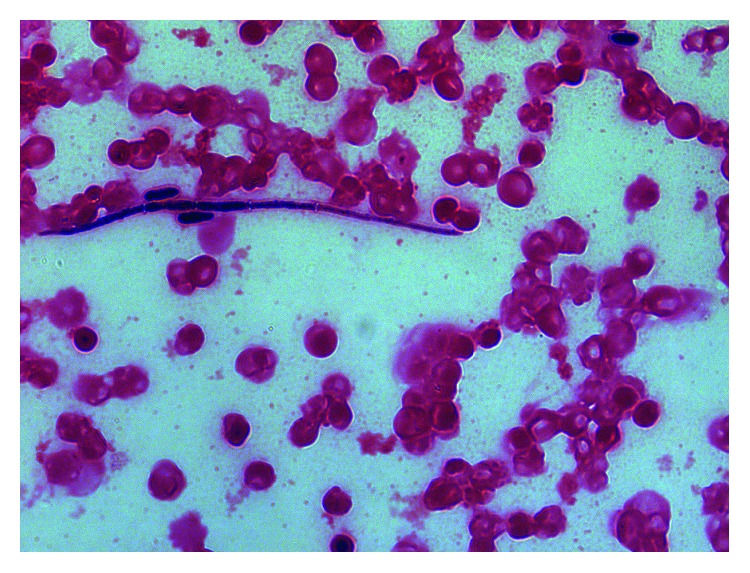
Gram stain from the blood culture bottle when the patient relapsed showing septate hyphae and ellipsoidal blastoconidium of *Acremonium*, mimicking pseudohyphae of candida.

A 21-year-old male with a history of Tetralogy of Fallot (TOF) repair (with Dacron Patch over a large ventricular septal defect (VSD)) was admitted with complaints of fever and weight loss for 2 months and left sided abdominal pain since 1 week. He was recently admitted with similar complaints to another hospital where he was found to have a right sided pneumonia and was being treated with intravenous ceftriaxone. Later, suspecting infective endocarditis, gentamicin was added but as the patient was still having persistent fevers of 40°C, he was referred to our hospital. On examination, the patient was of lean built with grade IV clubbing without cyanosis, and there were no peripheral stigmata of infective endocarditis. He had a loud pansystolic murmur on the left sternal edge and had tenderness on palpation of the left upper abdomen. His initial investigations showed a high white blood cell count and C-reactive protein (CRP) (Table 1). A chest X-ray showed left mid and right lower lung zone infiltrates, and an ultrasound of the upper abdomen showed an ill-defined splenic lesion without internal vascularity suggesting either an abscess or infarct. Three sets of blood cultures were subsequently negative. Echocardiogram showed vegetation on the VSD patch along with dehiscence, a large VSD, and moderate right ventricular outflow obstruction. A CT abdomen with contrast was done which showed multiple liver, splenic, and lung abscesses with infarcted left kidney and thrombus at the bifurcation of the aorta secondary to the septic embolic phenomenon. Cardiothoracic surgery consultation was sought, and the patient underwent a redo-sternotomy and removal of vegetations from right ventricular outflow tract site, removal of Dacron Patch, and complete repair of TOF. Postoperative echocardiogram did not show any residual VSD or vegetation, only mild left ventricular dysfunction and moderately reduced right ventricle function was seen. The vegetation removed from right outflow tract and Dacron Patch was sent for bacterial, mycobacterial, and mycology cultures. Chocolate, Sheep Blood (SBA), and MacConkey Agar plates were incubated at 37°C for aerobic culture and a SBA plate incubated anaerobically. For fungal culture, tissue was stabbed onto the surface of Sabouraud's dextrose (SDA), potato dextrose (PDA), and Mycosel agar plates incubated at 25°C and an additional SDA and SBA incubated at 37°C. The initial Gram stain and 10% potassium hydroxide (KOH) preparation revealed clusters of oval conidia and hyphae mistaken for yeast cells and pseudohyphae as well as septate hyphae. Patient was empirically started on intravenous amphotericin deoxycholate at 1 mg/kg (40 mg/dose), his fever gradually subsided, and the patient was discharged on amphotericin and oral voriconazole. Bacterial cultures remained negative, and a filamentous mould grew from the vegetation at 48 h that was finally identified phenotypically as *Acremonium* species (Figure 1). It grew on all plates except MacConkey agar. Mycobacterial cultures remained negative. Susceptibilities were performed by E-test® (bioMerieux, France) on RPMI agar, and the isolate showed an MIC of >32 mcg/ml against amphotericin and 2 mcg/ml against voriconazole. After final identification amphotericin, deoxycholate was discontinued and oral voriconazole 200 mg twice daily was continued. However, treatment was discontinued at 3 months based on clinical improvement and due to inability of the patient to bear the cost of the treatment. After 2 months of stopping therapy, patient had recurrence of symptoms along with new vegetation on the pulmonary valve. Blood cultures were submitted in BD BACTEC™ aerobic and anaerobic bottles and incubated in the BD BACTEC 9240 system, as BD BACTEC MYCO/F Lytic system was not available. The bottles flagged positive after 3 days of incubation and two sets of blood cultures grew *Acremonium* species with similar antifungal susceptibilities (Figure 2). The mould was identified on the colony and microscopic morphology. It was a hyaline mould appearing in 3 days on solid media, with cream coloured wrinkled surface and an off-white to tan reverse. By day 6, the colonies became slightly floccose. On microscopic examination, the hyphae were thin, hyaline, and septate, with short nonseptate conidiophores giving rise to elliptical conidia clinging to the philaides in wet masses of 10–20. Molecular confirmation could not be made; however, the isolate was phenotypically differentiated from *Fusarium* and *Phialemonium* species due to colony appearance, absence of macroconidia, and septations at the base of conidiophores.

Voriconazole was restarted and surgical excision of the pulmonary valve was performed two months later with growth of the similar fungus. The patient was continued on oral voriconazole for a period of one year with clinical improvement, improvement in inflammatory biomarkers, no residual vegetation on repeat echocardiogram, and serially negative blood cultures.

## 3. Discussion

A rare case of fungal infection of a VSD repair patch with *Acremonium* species is described. Diagnosis of this case was established by visualization of fungal elements on microscopy of vegetation tissue and later from culture. Relapse was confirmed by growth of a similar fungus in pulmonary valve tissue and two sets of blood cultures. Identification of all isolated fungi was determined by the conventional method. Although ITS region sequencing was performed on the blood culture isolates, it was not able to give reliable identification.

Although there have been reports of *Acremonium* species, particularly *Acremonium kiliense* causing peritonitis [6], involvement of lung and disseminated infection in immunocompromised patients [7], there are only few case reports of cardiac infection with *Acremonium* species (Table 2). In almost all cases, the infection was of a prosthetic valve or pacemaker [3, 8–10]. However, there have also been reports of native valve infective endocarditis with this organism [11, 12]. There have been several reports of fungemia usually associated with central line associated blood stream infection and mostly occurring in immunocompromised patients, but none complicated by associated endocarditis [2, 4, 13]. *Acremonium* endocarditis has resulted in mortality despite surgical treatment and intravenous amphotericin (Table 2). In a case of *Acremonium strictum* colonization of a prosthetic mitral valve, the patient died of multiorgan failure despite treatment with intravenous caspofungin and later voriconazole [9]. However, there have been reports of patients recovering successfully with azole treatment as well [3, 12]. The treatment duration has varied for both the cases that were treated successfully. Intravenous fluconazole at high doses was given for one month with success, and in another case, patient was treated with intravenous voriconazole initially and later with an oral formulation for six weeks duration and had an uneventful recovery [3]. Our patient was treated with surgical intervention as well as with voriconazole for three months initially but unfortunately relapsed and was subsequently treated for a whole year.

The case report that we have described is the first one from our region and unlike most other cases with adverse outcome, our patient recovered despite having advanced disease. Based on our review of other case reports as well as our own experience, we would recommend surgical intervention along with voriconazole therapy for a prolonged duration for better outcome in patients having *Acremonium* endocarditis.

## Figures and Tables

**Table 1:** Serial white blood cell count (WBC) and inflammatory markers erythrocyte sedimentation rate (ESR) and C-reactive protein (CRP).

Labs	On presentation	After 3 months of treatment	At relapse	After 1 year of treatment
WBC (×10^9^ per liter (L))	17.7	7.6	8.8	8.9
ESR (mm/hr)	65	14	87	18
CRP (mg/L)	22.1	<0.3	10.8	<0.1

**Table 2:** Summary of case reports of *Acremonium* endocarditis.

Case reports	This case	Panzaru et al. [12]	Guarro et al. [9]	Heitmann et al. [3]	Lacaz Cda et al. [10]	Degeorges et al. [11]
Age/gender	21 years/male	51 years/male	73 years/male	80 years/male	47 years/male	31 years/male

Risk factors	TOF repair with Dacron Patch over VSD	Immunocompetent, admitted with pneumonia and CHF	Severe COPD on steroids, prosthetic mitral valve	Had been on steroids for inflammatory eye disease, pacemaker	Prosthetic valve	Aortic stenosis

Species isolated	*Acremonium* spp.	*Acremonium* spp.	*Acremonium strictum*	*Acremonium* spp.	*Acremonium kiliense*	*Acremonium* spp.

Valve involved	No valves, VSD patch only	Pulmonary and tricuspid valves	Prosthetic mitral valve	Pacemaker related tricuspid valve	Prosthetic mitral valve	Aortic valve

Treatment given	Amphotericin deoxycholate and voriconazole for 3 months then voriconazole for 12 months, removal of Dacron Patch	Fluconazole 400 mg twice daily for 30 days	Caspofungin followed by voriconazole	Voriconazole for 6 weeks	Amphotericin B, 5 flucytosine, surgery	Amphotericin B

Outcome	Recovered	Recovered	Died	Recovered	Died	Died
